# Molecular Dynamics Simulation of a Jet in a Binary System at Supercritical Environment

**DOI:** 10.3390/molecules24010031

**Published:** 2018-12-21

**Authors:** Qingfei Fu, Zixuan Fang, Yunxiao Zhang, Lijun Yang

**Affiliations:** 1 School of Astronautics, Beihang University, Beijing 100191, China; fuqingfei@buaa.edu.cn (Q.F.); fzx2014good@163.com (Z.F.); yxzhang92@163.com (Y.Z.); 2 Beijing Advanced Innovation Center for Big Data-Based Precision Medicine, Beihang University, Beijing 100191, China

**Keywords:** molecular dynamics, supercritical environment, equation of state, growth rate

## Abstract

With the development of large-thrust liquid rocket engines, the behavior of liquid in supercritical conditions arouses increasing public interest. Due to the high pressure and temperature of the combustion chamber, fuel reaches its critical point much more easily, and enters supercritical conditions. Due to the drastic changes in the physical properties of the fluid near the critical point, it is usually difficult to simulate the fluid motion using traditional computational fluid dynamic methods; but molecular dynamics (MD) can simulate fluid motion at the molecular level. In view of the engineering application, the physical properties of a binary system consisting of argon and nitrogen, and the stability of subcritical jets sprayed into supercritical environment, has been studied here using the MD method. First, the molecular dynamic simulation of the equation of state (EOS) of the mixture was put forward. Four conditions, with different mixing ratios of nitrogen, were designed. The results showed that the mixing ratio of nitrogen noticeably affected the results; these results were compared with the Soave-Redich-Kwong (SRK) EOS. Second, a simulation was conducted of subcritical nitrogen jet sprayed into a supercritical argon environment. After analyzing the results, the jet density and temperature distributions were obtained and the disturbance growth rate of the shear layer was analyzed.

## 1. Introduction

The rapid development of aerospace technology and large-thrust liquid propellant rocket engines (LPRE) has brought with it increasing public attention. The spraying and mixing processes of fuels and oxidizers are generally the most important working steps in the rocket engine, greatly affecting the quality of atomization. Due to the high pressure and temperature in the combustion chamber, the jet of a fuel easily reaches a critical point and transforms into a supercritical state. Apart from rocket engine, supercritical jets are also used in many areas of daily life and industrial manufacture—such as supercritical fluid extraction—making the investigation and analysis of jet supercritical conditions of vital importance [1]. 

**Figure 1:**
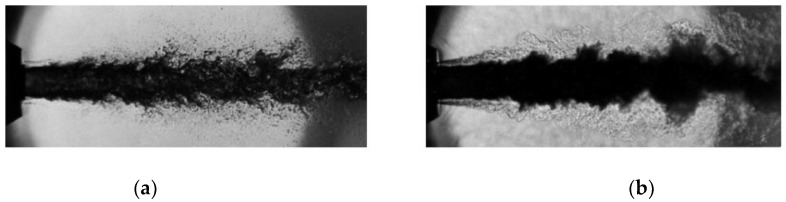
Shadowgraphs of coaxial liquid nitrogen injection with helium co-flow into helium environment at subcritical and supercritical pressure: (**a**) subcritical break-up, $p/p_{cr,N_{2}}=0.3$; (**b**) supercritical disintegration, $p/p_{cr,N_{2}}=1.8$ [2].

Unlike the traditional subcritical jet, properties of the jet near the critical point are much more complicated. Mayer found a significant difference between the subcritical and the supercritical jet [2]. As seen in Figure 1, when a subcritical jet is ejected into a subcritical environment, the instability of the jet surface increases with the growth of the jet. The surface of the jet breaks up and numbers of droplets can be observed. As the temperature and pressure increases, the environment enters into a supercritical state, where it can be observed that the interface between gas and liquid is replaced by mixing turbulence. According to Dahms and Oefelein [3], due to its high Knudsen number, the surface of a supercritical jet resembles a non-continuum molecular stream more than a conventional continuum regime, which can be defined as

(1) $$K_{n}=\frac{\lambda }{l}>0.1$$

Here, λ is the molecular mean free path, *l* is the corresponding interface thickness calculated from the linear gradient theory [4]. Traditional methods of solving subcritical jet problems are based on the continuum regime and Navier-Stokes equations, which do not easily explain supercritical phenomena.

Many studies have been performed [5,6,7,8,9,10,11,12,13] that focus on shear injection and mixing in supercritical environment using numerical methods. The molecular dynamics (MD) method and molecular dynamics simulations are based purely on first principles. If a calculation in physics begins directly at the level of the established laws of physics, and without assumptions such as empirical model and fitting parameters, it is said to be from *first principles*, or ab initio. Molecular dynamics simulations follow Newton’s second law at the molecular level and describe a molecule’s movement without any assumptions. As a result, the macro physical quantities can be obtained by statistical methods, a more realistic and useful approach for this research project. Chapela and Saville simulated the interface properties between gas and liquid argon by MD and compared the results with a Monte Carlo simulation (MC) [14]. They also adopted a hyperbolic tangent fitting to locate the interface. Nijmeijer and Bakker simulated the surface of Lenard-Jones liquids and compared experimental with theoretical result, finding a good fit [15]. Li-Jen Chen studied the relationship between the interface size and properties by MD and found that the size of interface had little impact on the liquid and gas density [16]. Moseler examined the information, stability and break-up processes by MD and proved that the MD method could accurately predict the development of a subcritical jet [17]. Shin investigated the phenomenon of the supercritical jet by MD, finding that the surface tension of a typical jet disappeared, with the layer of density clearly observable [18].

In the present study, an accurate simulation method of supercritical spray using MD was performed, and spatial instability of the single system jet mixing layer was studied. Zhang [19] simulated a system consisting of a trans critical nitrogen jet and a supercritical nitrogen environment, and found the spatial growth rate of jet. The present research simulated a binary system including subcritical nitrogen jet and a supercritical argon environment. The temperature and pressure of the argon environment were both over the critical point, to nitrogen. First, a binary system using MD methods was constructed and the calculating properties compared with theoretical results calculated using the Soave-Redlich-Kwong (SRK) equation of state. Then the molecular dynamics simulation of a nitrogen jet was performed at different conditions. The temperature and pressure of the environment was changed in an ascending order. Analysis showed the distribution of jet density, temperature and other jet characteristics. Finally, the growth rate of the shear layer under different conditions was defined and calculated.

## 2. Methodology

### 2.1. Molecular Dynamics Model

**Figure 2:**
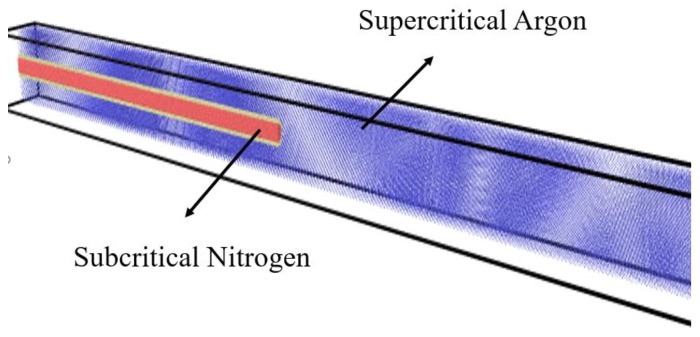
Closed binary system simulation box, size *L_x_* × *L_y_* × *L_z_* (*L_x_* = 200 Å, *L_y_* = 800 Å, *L_z_* = 20Å). A gold tube sits in the middle of the left surface, filled with subcritical nitrogen (120 K, 7.5 MPa, 454.09 kg/m^3^) and surrounded by supercritical argon (200 K, 5.5 MPa, 157.65 kg/m^3^).

**Figure 3:**
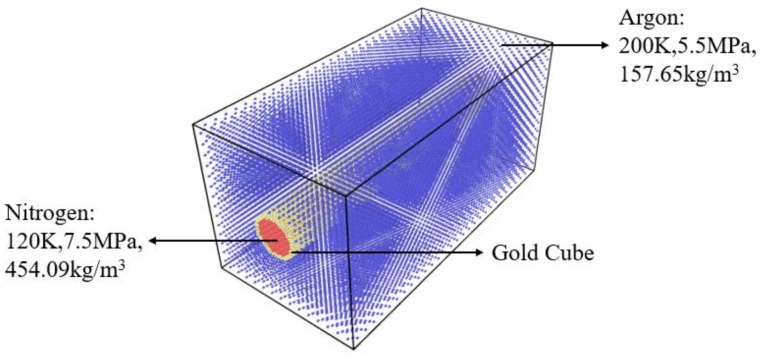
Left section of the simulation box, size *L_x_* × *L_y_* × *L_z_* (*L_x_* = 200 Å, *L_y_* = 400 Å, *L_z_* = 200 Å). A gold tube sits in the middle of the box, filled with subcritical nitrogen (120 K, 7.5 MPa, 454.09 kg/m^3^) and surrounded by supercritical argon (200 K, 5.5 MPa, 157.65 kg/m^3^).

**Figure 4:**
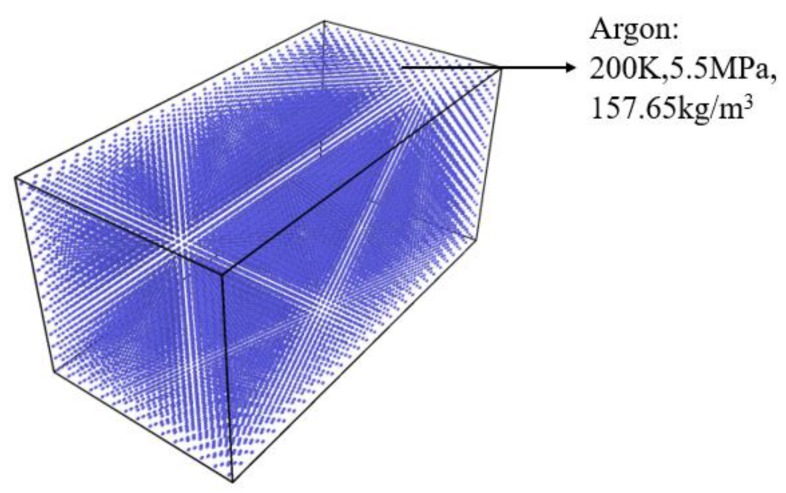
Right section of simulation box, size *L_x_* × *L_y_* × *L_z_* (*L_x_* = 200 Å, *L_y_* = 400 Å, *L_z_* = 200 Å). Box is filled with supercritical argon (200 K, 5.5 MPa, 157.65 kg/m^3^).

A closed binary system simulation box (Figure 2) was built to the size of *L_x_* × *L_y_* × *L_z_* (*L_x_* = 200 Å, *L_y_* = 800 Å, *L_z_* = 200 Å). A gold tube was fixed in the center left of the box. The lattice constant of gold was 4.08Å. Inside the tube was subcritical nitrogen (120 K, 7.5 MPa, 454.09 kg/m^3^). The initial diameter of the jet was 40Å. Outside, the tube was filled with argon (200 K, 5.5 MPa, 157.6 5 kg/m^3^) as the supercritical environment (Figure 3). The right section of the box was filled with supercritical argon (Figure 4). A periodic boundary condition (P.B.C.) was applied in x, y and z directions, respectively. About 34,172 nitrogen molecules, 43,066 argon molecules and 17,640 gold atoms were involved in the simulation; and the number of atoms changed under different working conditions.

This molecular dynamics simulation was performed using LAMMPS. LAMMPS stands for Large-scale Atomic/Molecular Massively Parallel Simulator; it is a classic molecular dynamics simulation code that focuses on materials modelling. It was designed to run efficiently on parallel computers. It was developed originally at Sandia National Laboratories, a US Department of Energy facility. [20]. After modeling, the simulation is implemented under an NVE ensemble to update all the nitrogen and argon molecules with a time step of 0.01 ps, using the classic parallel MD code [21]. Initial velocities of the nitrogen and argon molecules were determined randomly from the Gaussian distribution representative of a given initial temperature.

### 2.2. Force Routine

Each nitrogen molecule is composed of two nitrogen atoms, with an imaginary bond between them. Therefore, when nitrogen molecules were calculated for this work, the force within different molecules and the bond force were taken into account. Bond force does not need to be considered for argon molecules, due to their single-atom structure.

**Figure 5:**
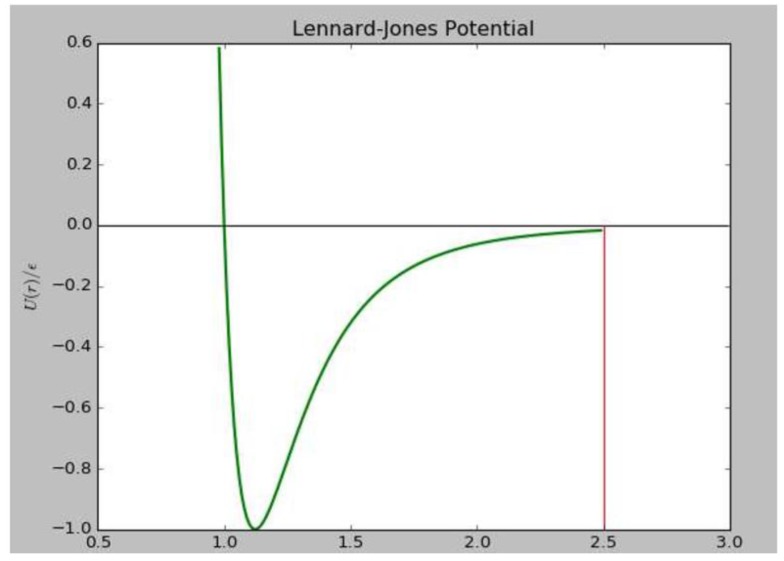
Leonard-Jones 12-6 potential.

The bond force can be expressed using a Harmonic potential:

(2) $$E(l)=K{\left(l-l_{b}\right)}^{2}$$

where *l* is the instant bond length of nitrogen molecular, $l_{b}=1.1\overset{\circ }{A}$ is the equilibrium bond length of nitrogen, *K* = 3.496 eV is the stretching force constant of the N≡N triple bond. The non-bond force between different molecules (also called the Van der Walls force), is described by Leonard-Jones(L-J) 12-6 potential:

(3) $$U_{LJ}(r)=4\varepsilon \left[{\left(\frac{\sigma }{r}\right)}^{12}-{\left(\frac{\sigma }{r}\right)}^{6}\right]$$

where *ε* is the potential well depth, *σ* is the core distance where potential equals to zero, *r* is the distance between two atoms. A cut off distance must be adopted to reduce the calculation. Once the distance between two molecules exceeds the cut off distance, the Van der Walls force can be ignored (Figure 5). It can be defined as:

(4) $$U_{trunc}(r)=\left\{ \begin{array}{ll} U_{LJ}(r), & r<r_{c}\\ 0, & r\geq r_{c} \end{array}\right.$$

Considering the hydrophobic and hydrophilic factors at the interface of liquid and tube, a fixed model is utilized to avoid the molecules accumulation:

(5) $$U_{LJ}(r)=4\alpha \varepsilon \left[{\left(\frac{\sigma }{r}\right)}^{12}-\beta {\left(\frac{\sigma }{r}\right)}^{6}\right]=4\varepsilon^{\prime }\left[{\left(\frac{\sigma^{\prime }}{r}\right)}^{12}-{\left(\frac{\sigma^{\prime }}{r}\right)}^{6}\right]$$

(6) $$\left\{ \begin{array}{l} \sigma^{\prime }=\sigma /\beta^{1/6}\\ \varepsilon^{\prime }=\alpha \beta^{2}\varepsilon \end{array}\right.$$

where α is hydrophilic parameter, *β* is hydrophobic parameter, $\sigma^{\prime }$ and $\varepsilon^{\prime }$ are actual parameters for solid-liquid interaction. Specific values of these symbols are given by Zhang [19].

### 2.3. Equation of State

During the temperature transition across the critical value (especially when pressure approaches the critical point), thermodynamic and transport anomalies may occur [22], the ideal gas equation of state (EOS) is no longer applicable. Therefore, a real gas EOS, which includes the effects of true physics, must be considered. Soave raised a new concept, called the eccentricity factor, which was only attached to the structure of the molecules [23]. Soave also modified Redich-Kwong EOS with the eccentricity factor and dimensionless temperature, focusing on the square root item:

(7) $$\alpha ={\left(1+(0.48508+1.55171\omega -0.15613\omega^{2})(1-T_{r}^{0.5})\right)}^{2}$$

where *w* is the eccentricity factor, $T_{r}=T/T_{c}$ is the dimensionless temperature, ratio of the temperature and its critical value. The real gas EOS becomes:

(8) $$p=\frac{RT}{V-b}-\frac{a\alpha }{V(V+b)}$$

where *p* is pressure, *R* is the universal gas constant, *V* is molecular volume, *a* and *b* are adjustable parameters:

(9) $$a=\frac{0.42747R^{2}T_{c}^{2}}{p_{c}}$$

(10) $$b=\frac{0.08664RT_{c}}{p_{c}}$$

Compared with traditional R-K EOS, SRK EOS takes the eccentricity factor and dimensionless temperature into account. It has been proved that SRK EOS can imitate real gas properties in a wide range.

To this point, the availability of SRK EOS in single-component system has been examined; but SRK EOS is also applicable in a binary system [23]. When applied in a binary system, the critical temperature and pressure value in SRK EOS changes, depending on the mixing ratio of each component:

(11) $$T_{cm}=\sum x_{i}T_{ci}$$

(12) $$p_{cm}=\sum x_{i}p_{ci}$$

where, *x_i_* is the mixing ratio of each component, *T_ci_* and *p_ci_* are the critical temperature and pressure of each component, *T_cm_* and *p_cm_* are the actual critical temperature and pressure of the binary system.

Experiment shows that only when the temperature and pressure ratios of each component range from 0.5 to 2.0, can the calculated compressibility factor be more accurate [24]. It can be described as:

(13) $$0.5<T_{cr,i}/T_{cr,j}<2,\;0.5<p_{cr,i}/p_{cr,j}<2$$

In this simulation, the critical point was 126 K and 3.38 MPa for nitrogen and 151 K and 4.86 MPa for argon. The application condition of the binary system SRK EOS can be well satisfied.

### 2.4. Spatial Growth Rate

Charpentier’s method was followed to obtain the growth rate of the liquid spray [25]. As is known, Rayleigh proposed the first model of inviscid jet using a linear stability theory [26]. In this theory, the evolution of a single mode perturbation to an infinite column of liquid can be described by:

(14) $$\eta (y,t)=\eta_{0}\sin(ky+\phi )e^{\alpha t}$$

where *y* is the position of the jet axis, *t* the time, *k* the wavenumber of the perturbation, ϕ the spatial phase, *α* the growth rate and $\eta_{0}$ the initial amplitude of the perturbation. The jets are transported with a uniform velocity *U* so the temporal and spatial variables are linked by *y* = *Ut*.

For a set of jet profiles obtained by MD, the radius of jet at each axis position *y* is

(15) $$r_{i}(y)=\frac{d}{2}+\eta_{0}\sin(ky+\phi_{i})e^{\alpha y/U}$$

where *d* is the diameter of jet, and $\phi_{i}$ is the perturbation spatial phase of the $i^{th}$ profile. The equation is then turned dimensionless, using diameter as the length scale and Rayleigh time as the time scale:

(16) $$t_{R}=\sqrt{\left(\rho d^{3}\right)/\left(8\gamma \right)}$$

with *ρ* and *γ* the density and surface tension, respectively.

The dimensionless result obtained is:

(17) $$\bar{r_{i}}(\bar{y})=\frac{1}{2}+\bar{\eta_{0}}\sin(\bar{k}\bar{y}+\phi_{i})e^{\bar{\alpha }\bar{y}/\bar{U}}$$

where $\bar{y}=y/d$, $\bar{k}=kd$, $\bar{\alpha }=\alpha t_{R}$, $\bar{U}=Ut_{R}/d$, $\bar{\eta_{0}}=\eta /d$, $\bar{\eta_{i}}=\eta_{i}/d$. The standard deviation is computed at each *y* axis position as

(18) $$\bar{\sigma }(\bar{y})=\frac{1}{\sqrt{N}}\sqrt{\sum_{i=1}^{N}{\left(\bar{r_{i}}(\bar{y})-\langle \bar{r_{i}}(y)\rangle \right)}^{2}}$$

where $\langle \bar{r_{i}}(\bar{y})\rangle$ is the expected value of $\bar{r_{i}}(y)$ over *i* at the position *y*. Assuming that the jet profiles are not correlated in time, each phase $\phi_{i}$ is modeled as the realization of a random variable of uniform distribution on [0, 2π]. So $\langle \bar{r_{i}}(\bar{y})\rangle$ tends to 0.5 when N is sufficiently large. The equation becomes

(19) $$\bar{\sigma }(\bar{y})=\frac{1}{\sqrt{N}}\sqrt{\sum_{i=1}^{N}{\left(\bar{\eta_{0}}\sin(\bar{k}\bar{y}+\phi_{i})e^{\bar{\alpha }\bar{y}/\bar{U}}\right)}^{2}}$$

Using the uniform distribution of the random variable defining $\phi_{i}$ and taking the logarithm of Equation (13), we finally obtain

(20) $$\ln(\bar{\sigma }(\bar{y}))=\ln(\frac{\left|\bar{\eta_{0}}\right|}{\sqrt{2}})+\frac{\bar{\alpha }}{\bar{U}}\bar{y}$$

This formula associates the standard deviation of the radius at each position $\bar{\sigma }(\bar{y})$ with the spatial growth rate $\bar{\alpha }$. This formula was used here to obtain the spatial growth rate of nitrogen jet perturbation.

## 3. Results and Discussion

### 3.1. Equation of State

**Figure 6:**
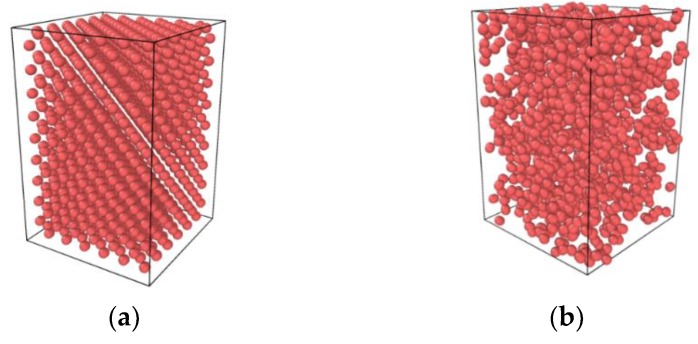
Nitrogen model made by molecular dynamics: (**a**) initial nitrogen distribution; (**b**) well-proportioned nitrogen.

**Figure 7:**
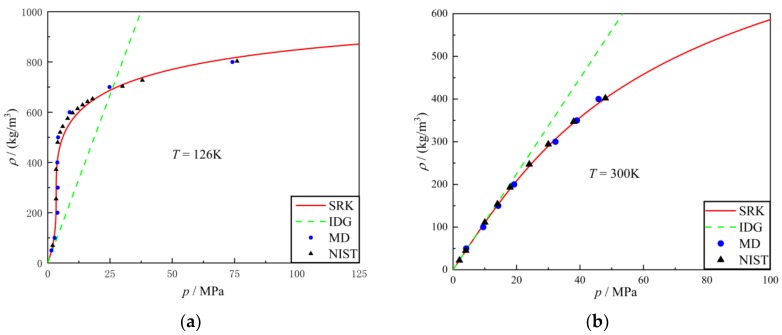
Results of molecular dynamics of nitrogen compared with SRK EOS, ideal gas EOS and NIST data at different temperatures: (**a**) *T* = 126 K; (**b**) *T* = 300 K.

**Figure 8:**
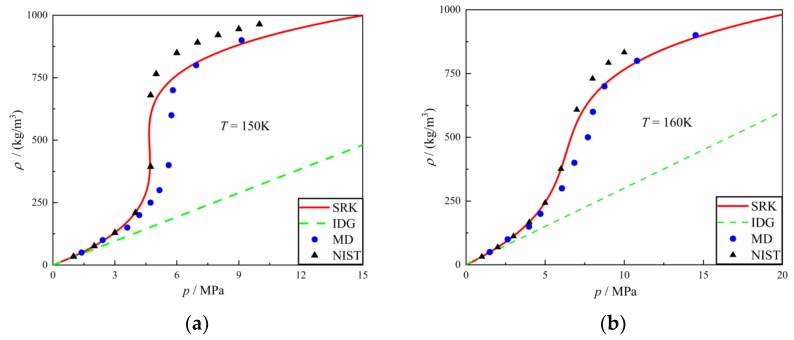
Results of molecular dynamics of argon compared with SRK EOS, ideal gas EOS and NIST data at different temperatures: (**a**) *T* = 150 K; (**b**) *T* = 160 K.

Zhang simulated the thermodynamic properties of nitrogen using MD and compared the results with SRK EOS [19]. Referencing his method, a dimensional result was simulated using MD. As shown in Figure 6, a finite regime was constructed and nitrogen molecules of a specified density were aligned to the grid. This one-component system was given an NVE ensemble and imported into simulation software. After a given time, the software calculated the pressure of each specified density and the data was compared with IDG (ideal gas) EOS, SRK EOS and NIST. The result is shown in Figure 7. The blue spot is the result from MD; the solid red line is the SRK equation of state. The dashed green line is the ideal gas equation of state, for reference purposes. It is apparent that the state equations obtained by molecular dynamics simulation are perfectly in line with the SRK EOS and NIST. Note that, the properties of argon were simulated using MD at different temperatures and the results compared with IDG EOS, SRK EOS and NIST in Figure 8.

**Figure 9:**
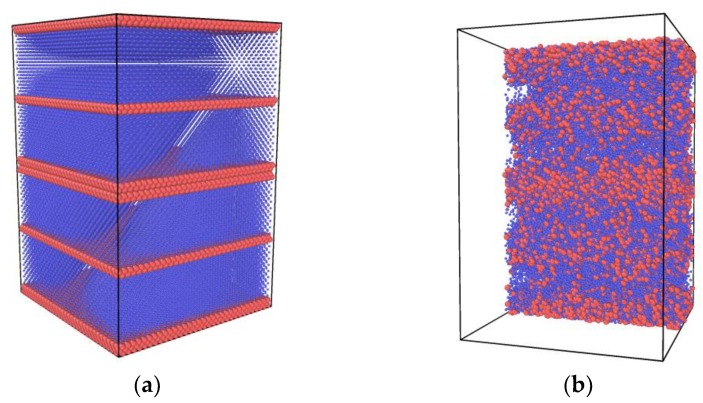
A new *sandwich* molecular dynamics model: (**a**) Initial system distribution; (**b**) well-proportioned system.

**Figure 10:**
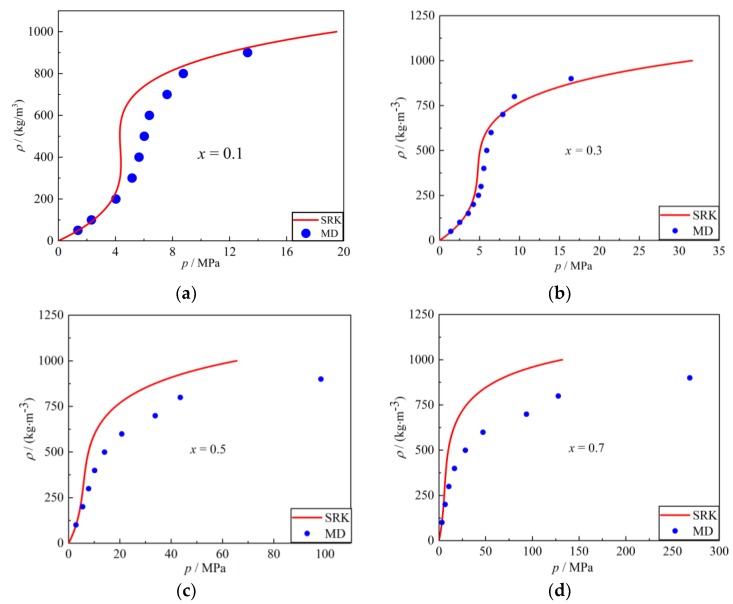
Results of molecular dynamics comparing SRK EOS with different mixing ratio of nitrogen: (**a**) *x* = 0.1; (**b**) *x* = 0.3; (**c**) *x* = 0.5; (**d**) *x* = 0.7.

The most obvious difference between the one-component system and the binary system is the mixing ratio of each component; here, the mixing ratio of nitrogen in mole was taken as a parameter. In the simulation process, it is often difficult for the two components to mix uniformly. To solve this problem, a *sandwich* model was adopted (Figure 9). The nitrogen and argon molecules were arranged in layers-like a sandwich. Using this model, the binary system could be uniformly mixed. A specified nitrogen mixing ratio was set and the results are shown in Figure 10. The blue spot is the result from the MD, while the solid red line is the SRK equation of state. It can be concluded that as the mixing ratio of nitrogen increased, the accuracy of the binary system model decreased. The nitrogen molecule is composed of two atoms, argon has one, indicating that as the ratio of nitrogen increased, the truncation distance of the system became more complicated. It is difficult to solve this dilemma completely. Therefore, the mixing ratio of nitrogen molecules of the spray simulation system was limited to less than 0.3.

### 3.2. Nitrogen Jet Profile

**Figure 11:**
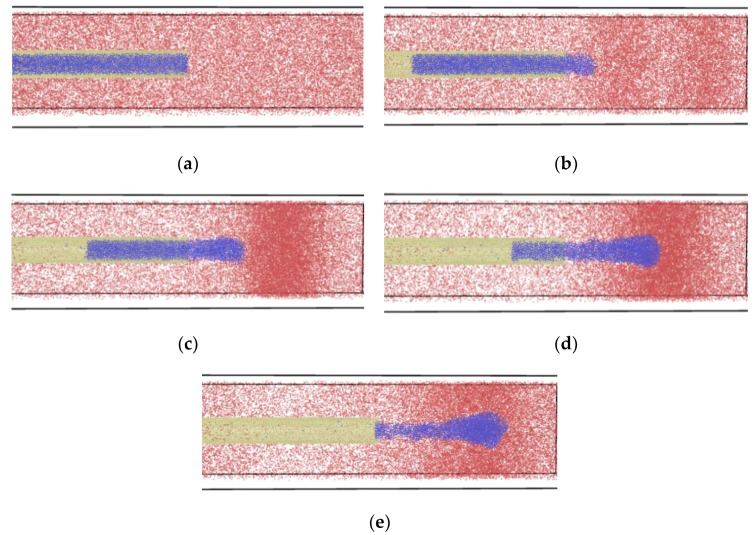
Development of subcritical nitrogen jet (7.5 MPa, 120 K) sprayed into supercritical argon environment (5.5 MPa, 200 K): (**a**) t = t_0_; (**b**) t = t_0_ + 25 ps; (**c**) t = t_0_ + 50 ps; (**d**) t = t_0_ + 75 ps; (**e**) t = t_0_ + 100 ps.

The result of molecular dynamics simulation of the subcritical nitrogen jet is visualized in Figure 11. The temperature and pressure of the nitrogen jet were 120 K and 7.5 MPa, while the argon environment was 200 K and 5.5 MPa. It is notable that the temperature and pressure were over the critical point of the nitrogen, as well as the argon. The original jet molecules are in blue; the ambient gas molecules are red. From the series of jet images, many characteristics of the subcritical jet and its transforming process are visible. At the beginning of the process, the jet was still in subcritical condition and its diameter remained unchanged. After some time, it was observed that the liquid jet absorbed heat from environment and swelled slightly, and a clear vapour-liquid interface appeared. Finally, the jet totally entered the ambient gas. The perturbation wave on the surface of the jet was easily observable.

Next, the series images will be explained in detail and the destabilization mechanism of subcritical jets will be clarified.

**Figure 12:**
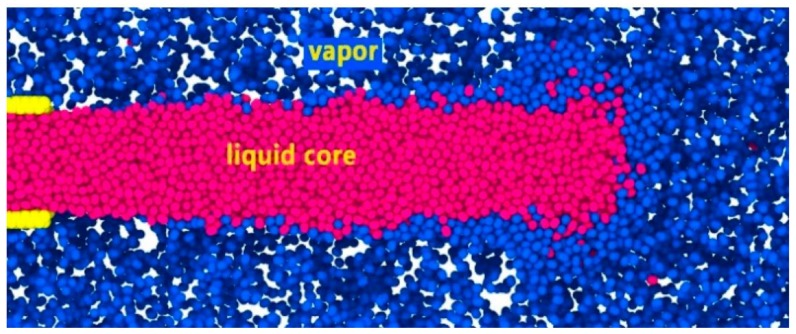
Two-dimensional (2-D) axial sectional view of subcritical jet [19].

According to Zhang [19], there is no theoretical difference between gas-liquid two-phase in supercritical fluid. Since the initial temperature of the fuel is lower than the critical temperature, the jet does not immediately reach the supercritical state after it is injected into the supercritical environment, but under the action of heat transfer, the jet gradually transforms into a supercritical state. Therefore, the transcritical/supercritical liquid jet structure consists of a liquid core and a thick vapor-liquid mixing layer surrounding the liquid core and an external high-density gas. The mixing layer is a transitional layer from the liquid state transition to the gaseous state, in which there is a large density gradient. No droplets, but scattered nitrogen vapor molecules exit in the system. After the diameter of the liquid core reduces to zero, the jet is completely atomized. The liquid core is defined in Figure 12.

**Figure 13:**
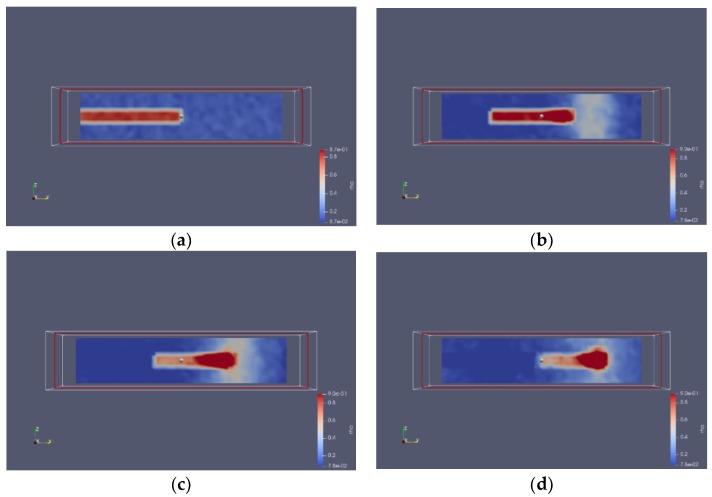
Density profile (unit: kg/m^3^) of nitrogen jet (7.5 MPa, 120 K) sprayed into supercritical argon environment (5.5 MPa, 200 K): (**a**) t = t_0_; (**b**) t = t_0_ + 25 ps; (**c**) t = t_0_ + 50ps; (**d**) t = t_0_ + 100 ps.

**Figure 14:**
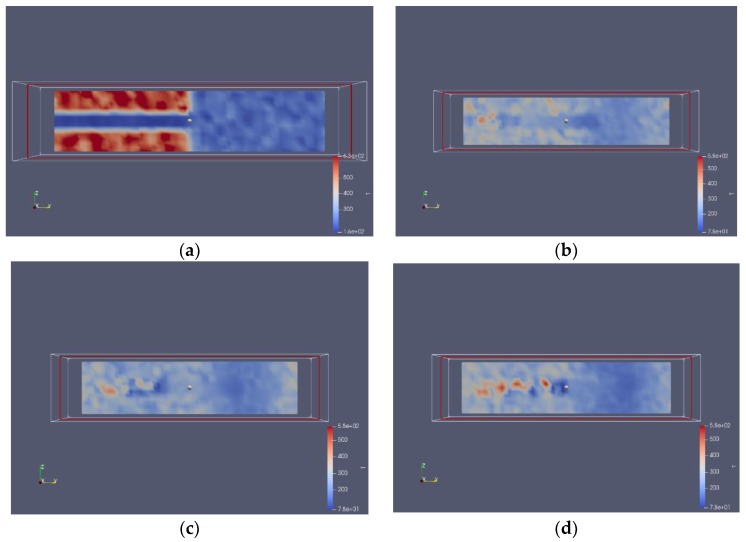
Temperature profile (unit: K) of nitrogen jet (7.5 MPa, 120 K) sprayed into supercritical argon environment (5.5 MPa, 200 K): (**a**) t = t_0_; (**b**) t = t_0_ + 25 ps; (**c**) t = t_0_ + 50 ps; (**d**) t = t_0_ + 100 ps.

According to previous researchers [27,28], the density profile should be determined prior to the instability analysis; therefore, both density and temperature profiles were obtained here using specified methods. The profiles of different times are shown in Figure 13 and Figure 14. A thin boundary layer can be clearly observed from the density profiles, proving that the subcritical jet molecules have mixed with the supercritical environment molecules in the boundary.

### 3.3. Spatial Growth Rate

**Figure 15:**
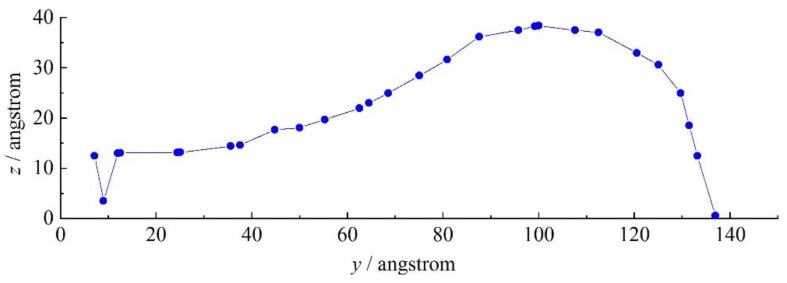
Contour of nitrogen jet (7.5 MPa, 120 K) sprayed into supercritical argon environment (5.5 MPa, 200 K) at t = t_0_ + 100 ps.

To obtain the growth rate of disturbances, an artificial gas-liquid interface should be determined by the blending rate of jet and ambient gas. We extracted the contour of the subcritical jet, as shown in Figure 15. It can be observed that after leaving the tube, the subcritical jet spread in all directions and formed a stable flow. There was a trend toward breakage near the tube exit.

**Figure 16:**
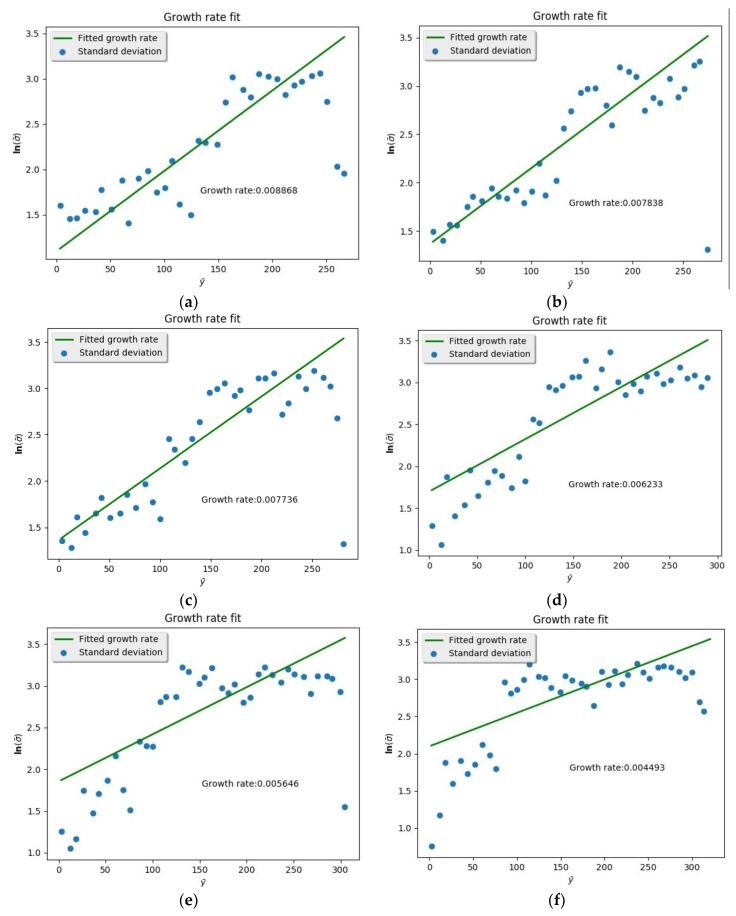
Spatial growth rate at different environmental pressure (all environmental pressures were over the critical point to the nitrogen and argon): (**a**) *p* = 6.5 MPa; (**b**) *p* = 7.5 MPa; (**c**) *p* = 8.5 MPa; (**d**) *p* = 9.5 MPa; (**e**) *p* = 10.5 MPa; (**f**) *p* = 11.5 MPa.

**Figure 17:**
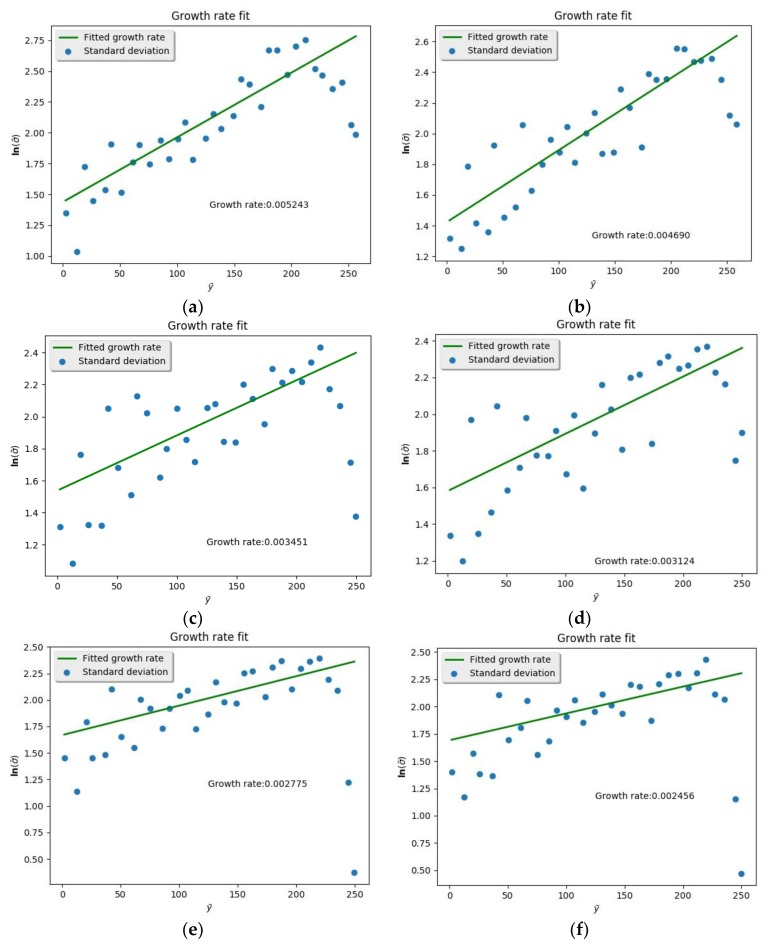
Spatial growth rate at different environmental temperatures (all environmental temperatures were over the critical point to the nitrogen and argon): (**a**) *T* = 300 K; (**b**) *T* = 400 K; (**c**) *T* = 500 K; (**d**) *T* = 600 K; (**e**) *T* = 700 K; (**f**) *T* = 800 K.

**Figure 18:**
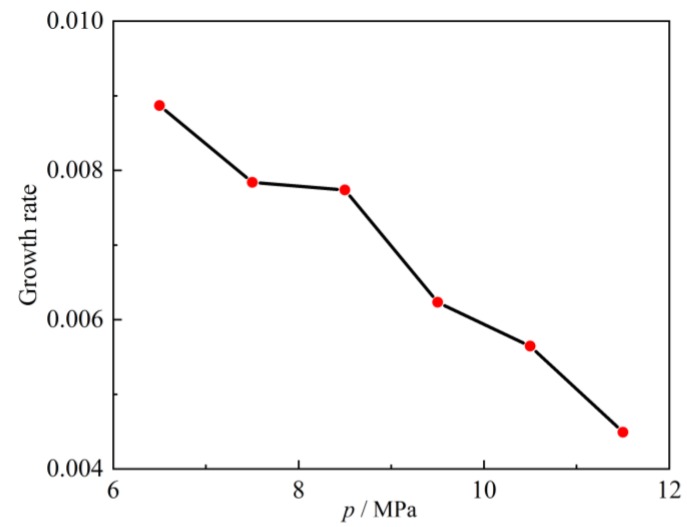
Growth rate curve with environmental pressure increases from 6.5 MPa to 11.5 MPa.

**Figure 19:**
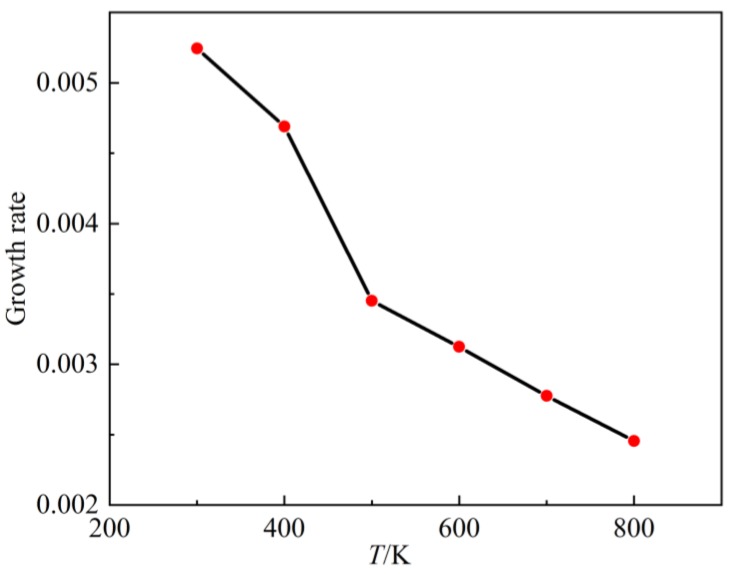
Growth rate curve with environment; temperature increases from 300 K to 800 K.

After finding the contour, the pressure and temperature of the argon environment were changed in ascending order and the spatial growth rate calculated, respectively (Figure 16 and Figure 17). Spatial growth rates under various conditions were collected and drawn into curves (Figure 18 and Figure 19). Figure 17 shows the variation of spatial growth rate when the argon pressure changed. From the figure it can be seen that the growth rate decreased as the pressure of the environment increased, so that the nitrogen jet became more and more stable. Figure 19 shows the variation of spatial growth rate when the temperature of the argon changed; it can be seen that the growth rate decreased rapidly in some temperature sections and then decreased slowly in some sections as the temperature of the environment increased, meaning that a temperature section existed where the nitrogen jet was most unstable. Zhang calculated the spatial growth rate of the nitrogen jet sprayed into different conditions of nitrogen environment [19]. Due to the different simulation systems (pure nitrogen system and binary system), the results of the simulation displayed some difference. The relative molecular mass of argon was larger than the mass of nitrogen and noticeably limited the diffusion of the jet, so it is reasonable that the spatial growth rate decreased when a subcritical jet sprayed into the argon environment, rather than into the nitrogen environment.

## 4. Conclusions

In this paper, the molecular dynamics method was utilized to study a binary system composed of subcritical nitrogen and supercritical argon. First, the thermodynamic property of the two-component system was obtained by molecular dynamics simulation and found to be in good agreement with the Soave-Redich-Kwong (SRK) equation of state, proving the accuracy of the model using the molecular dynamics method. It was also found that the model became increasingly accurate as the ratio of nitrogen decreased. Second, the density and temperature profile of a subcritical nitrogen jet ejected into a supercritical argon environment was obtained by molecular dynamics simulation. Finally, the spatial growth rate of the surface perturbation was obtained by fitting it to the growth rate formula. The growth rate decreased as the environmental pressure increased, while some sections decreased rapidly and then decreased slowly as the temperature of the environment increased. This results should provide reliable guidance for future study.
